# *Hes6* Is Required for the Neurogenic Activity of Neurogenin and NeuroD

**DOI:** 10.1371/journal.pone.0027880

**Published:** 2011-11-16

**Authors:** Kasumi Murai, Anna Philpott, Philip H. Jones

**Affiliations:** 1 MRC Cancer Cell Unit, Hutchison-MRC Research Centre, Addenbrooke's Hospital, Cambridge, United Kingdom; 2 Department of Oncology, University of Cambridge, Hutchison-MRC Research Centre, Addenbrooke's Hospital, Cambridge, United Kingdom; Institute of Science and Technology Austria, Austria

## Abstract

In the embryonic neural plate, a subset of precursor cells with neurogenic potential differentiates into neurons. This process of primary neurogenesis requires both the specification of cells for neural differentiation, regulated by Notch signaling, and the activity of neurogenic transcription factors such as neurogenin and NeuroD which drive the program of neural gene expression. Here we study the role of Hes6, a member of the hairy enhancer of split family of transcription factors, in primary neurogenesis in *Xenopus* embryos. *Hes6* is an atypical *Hes* gene in that it is not regulated by Notch signaling and promotes neural differentiation in mouse cell culture models. We show that depletion of *Xenopus* Hes6 (Xhes6) by morpholino antisense oligonucleotides results in a failure of neural differentiation, a phenotype rescued by both wild type Xhes6 and a Xhes6 mutant unable to bind DNA. However, an Xhes6 mutant that lacks the ability to bind Groucho/TLE transcriptional co-regulators is only partly able to rescue the phenotype. Further analysis reveals that Xhes6 is essential for the induction of neurons by both neurogenin and NeuroD, acting via at least two distinct mechanisms, the inhibition of antineurogenic Xhairy proteins and by interaction with Groucho/TLE family proteins. We conclude Xhes6 is essential for neurogenesis *in vivo*, acting via multiple mechanisms to relieve inhibition of proneural transcription factor activity within the neural plate.

## Introduction

During development, neural specification delineates the neural plate from the surrounding ectoderm that is destined to form epidermis. Whilst all early neural plate progenitor cells are competent to undergo neurogenesis, only a subset actually exit from the cell cycle and differentiate into neurons, a process that is controlled by the expression and activity of proneural proteins. The generation of primary neurons, the first neurons to differentiate within the neural plate, has been studied extensively in neurula *Xenopus* embryos, where the primary neurons expressing the differentiation marker neural ß tubulin (N-tubulin) are generated in three distinct domains on either side of the midline [1], [2].

A key step in neurogenesis is expression and activity of the basic helix-loop-helix proneural transcription factors that both specify the neuronal lineage and drive neuronal differentiation. The neurogenic transcriptional program of *Xenopus* primary neurons depends on the sequential activation of proneural proteins of the Atonal/Neurogenin family, neurogenin (Xngn2, also known as Xngnr1 in *Xenopus*) and NeuroD, which heterodimerize with ubquitously expressed E proteins to activate transcription [3], [4], [5], [6]. Neurogenin induces the transcription of a range of target genes implicated in neurogenesis [7], and is required for neural commitment in *Xenopus*, Zebra Fish and mouse, as when the protein is depleted or absent cells that would normally form neurons adopt glial fate [8], [9], [10]. Conversely, overexpression of Neurogenin drives cells into the neural lineage in *Xenopus*, chick and rat [3], [11], [12]. NeuroD is a central effector of Neurogenin function, sharing a number of common transcriptional targets in *Xenopus* and mouse [7]. NeuroD is also able to promote ectopic neurogenesis when mis-expressed in *Xenopus*, but has a more restricted neuronal phenotype in knockout mice [4], [13].

Maintaining the balance between progenitor maintenance and differentiation is essential for generation of the appropriate number of neurons at different developmental stages. One key pathway regulating this balance is downstream of the Notch receptor [2]. Notch acts via downstream effectors including members of the Hes family of transcription factors, such as *Xhairy1, 2A* and *2B* in *Xenopu*s and *Hes1* and *Hes5* in mammals [14], [15], [16], [17]. These Notch regulated Hes genes are key negative regulators of neural differentiation. Over expression of *Xhairy* in *Xenopus* or *Hes1* in mice blocks neuron formation [18], [19]. In contrast, loss of *Hes1* results in premature neuronal differentiation, and mice null for both *Hes1* and *Hes5* are refractory to the inhibitory effects of Notch signaling on neurogenesis [20], [21]. Recently it has been shown that *Hes1* expression oscillates in antiphase with *neurogenin 2* expression in neural precursor cells, commitment to terminal differentiation resulting in sustained repression of *Hes1* expression and upregulation of neurogenin [22].

**Figure 1:**
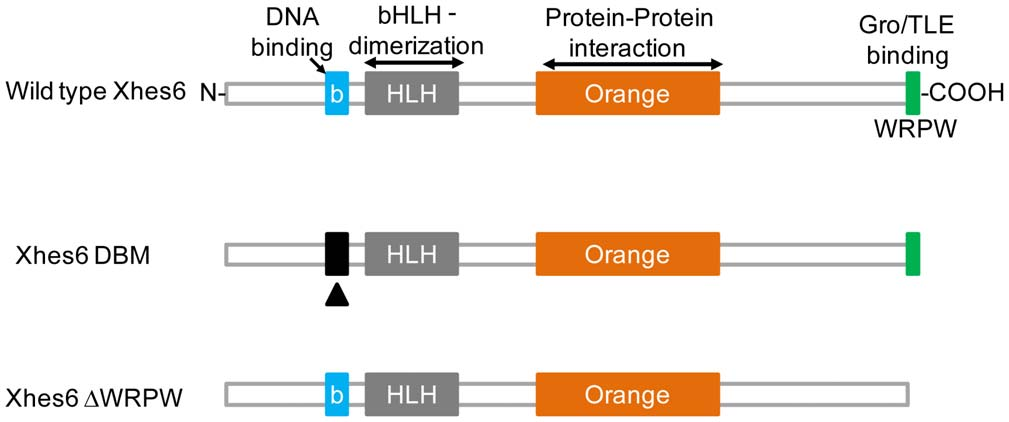
Conserved domains in Xhes6 and mutants used in this study. Xhes6 contains a conserved basic domain, required for DNA binding, a helix-loop-helix domain, implicated in dimerization with Xhes6 and other bHLH proteins, an “orange” domain, comprising the third and fourth helices of the protein, required for protein-protein interaction, and a C terminal WRPW motif, required for binding to Groucho/TLE family transcriptional coregulatory proteins. In this study a mutant of the basic domain which is unable to bind DNA (Xhes6 DBM) and a mutant lacking the WRPW motif (Xhes6 ΔWRPW) were used.

Here we focus on the role of another Hes family protein, *Hes6* in primary neurogenesis. *Hes6* is distinctive in that it is not regulated by Notch, lies downstream of Neurogenin, and promotes neurogenesis when overexpressed in *Xenopus*, cultured mouse neural progenitors or retinal explants [23], [24], [25]. The protein shares four highly conserved domains with other Hes proteins: a basic domain required for DNA binding, a Helix loop helix domain required for protein dimerization, an orange domain by which it binds to other Hes proteins and a C-teminal WRPW motif that recruits the Groucho/TLE family transcriptional corepressor proteins (Fig. 1) [26]. The sequence of the Hes6 loop domain is distinct from other Hes proteins giving it distinctive DNA binding properties compared to the Notch regulated Hes proteins [23], [27]. One potential mechanism whereby Hes6 promotes neurogenesis has been proposed to be binding to the anti-neurogenic, Notch regulated Hes proteins. For instance, in the mouse, Hes6 binds to Hes1, both preventing Hes1 from binding DNA and destabilizing the Hes1 protein [23], [24], [25]. In chick spinal cord there are two cHes6 genes, which act to repress both the transcription and function of *Hes5* [28], [29]. Knockdown of mouse Hes6 in primary cultures of mouse dorsal telencephalon produced a decrease in the proportion of NeuN positive cells and a larger increase in the proportion of cells exhibiting an astrocytic morphology and expressing the astrocyte marker protein GFAP [30]. However, it is unclear from this study whether cells were diverted from neural to glial fate or whether the differences seen reflect differences in survival and/or proliferation of lineage committed progenitors. In contrast over expression of Hes6 inhibits glial differentiation *in vitro*, a function it shares with neurogenin [11], [30], [31].

Collectively, the published work on Hes6 argues that it has a conserved role in promoting vertebrate neurogenesis, interacting with neurogenin and blocking the action of the antineurogenic, notch regulated Hes genes. We set out to explore these crucial interactions in the well characterized system of neural plate stage *Xenopus* embryos, which can integrate findings from disparate cell and tissue studies in a well characterized and accessible *in vivo* model of vertebrate development. By using antisense morpholino oligonucleotides to deplete *Xenopus* Hes6 (Xhes6) we demonstrate it is essential for neurogenesis early *Xenopus* embryos. We further show that Xhes6 is required for the induction of neurons by both Xngn2 and NeuroD, acting via at least two distinct mechanisms, the inhibition of antineurogenic Xhairy proteins and by interaction with Groucho/TLE family proteins. These observations reveal Xhes6 as an essential protein for neurogenesis in the early embryo, where it acts to promote the function of proneural transcription factors by multiple mechanisms.

## Results

### Expression of *Xhes6*, *Xhairy1* and *Xgrg4* in *Xenopus* neurula stage embryos

We began by confirming the expression of pattern *Xhes6* mRNA and transcipts encoding the proteins with which it interacts, *Xhairy1* and *Xgrg4* (Fig. S1). Consistent with previous reports, we find that *Xhes6* is expressed strongly in the posterior region of neurula stage embryos, but is also present in the medial and lateral domains of the neural plate and at low levels anteriorly (Fig. S1, [24]). The expression of *Xhairy1* is both more restricted and clearly delineated than that of *Xhes6*, lying in fine stripes in neural plate and also in the trigeminal ganglia and placode areas (Fig. S1). Groucho/TLE transcriptional cofactors are expressed widely in early stage embryos, but their expression becomes more restricted during development [32], [33]. We detected transcripts of *Xgrg2* and *Xgrg4* within and around the neural plate in neurula stage embryos (Fig. S1,data not shown). Thus at neural plate stage, *Xhes6*, *Xgrg2* and Xgrg*4* and *Xhairy1* each have a distinctive pattern of expression, but are all expressed within the neural plate.

### Xhes6 is required for neuronal differentiation

**Figure 2:**
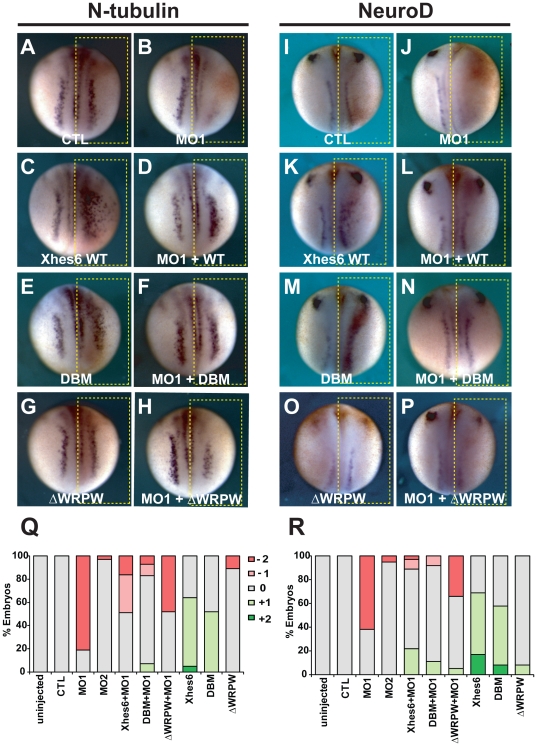
Xhes6 expression is required for neural differentiation. Whole mount *in situ* hybridization of neurula stage embryos for neural markers *neural* β*-tubulin* (*N-tubulin*, A-H) or *NeuroD* (I-P). A, B, I, J: One cell of two cell stage embryos was injected with control (CTL, A, I) or Xhes6 (MO1, B, J) morpholinos along with β-gal tracer mRNA (red staining). Injection of MO1 decreases both *N-tubulin* and *NeuroD* expression on the injected side (yellow box). C-H, K-P: Rescue of MO1 phenotype. 500 pg of mRNA encoding wild type Xhes6 or DNA binding domain (DBM) or Groucho binding domain, (ΔWRPW) mutants was injected with or without MO1. Overexpression of Xhes6 (C, K) or DBM (E,M) alone enhances neural differentiation, both Xhes6 and DBM rescue neural maker expression in Xhes6 morphants (D, F, L, N). The ΔWRPW mutant has minimal effect when injected alone (G, O) and is less efficient in rescuing the MO1 phenotype. Q,R quantitation of phenotypes observed, scored as in Fig. S2, Q, *N-tubulin* mRNA expression, corresponding to panels A-H, R, *NeuroD* mRNA expression, corresponding to panels I-P. Full data on the frequency of phenotypes and the number of embryos analyzed is given in Table 1.

To examine whether Xhes6 is required for primary neurogenesis, we used previously validated antisense morpholino oligonucleotides to prevent translation of *Xhes6* mRNA, [33]. *Xenopus* embryos were injected with either a control morpholino (CTL) or morpholinos against Xhes6 (Xhes6 MO1) in one cell at two-cell stage and analysed for the expression of the early neural progenitor marker *Sox3*, *NeuroD* and *Neural beta-tubulin* (*N-tubulin*), a marker for terminally differentiated primary neurons, at neurula stage, comparing the imjected and the uninjected sides. Scoring followed the scheme shown in Figure S2. There was no change in the expression of any of these markers in embryos injected with CTL (Fig. 2A, 2I, 2Q, 2R and Table 1). Injection of Xhes6 MO1 had no effect on *Sox3* expression (data not shown) but markedly reduced expression of both *N-tubulin* (in 81% of embryos (n = 31, Fig. 2B, 2Q and Table 1) and *neuroD* (in 62% of embryos, n = 39, Fig. 2J, 2R and Table 1). To confirm that the inhibition of primary neurogenesis was caused specifically by loss of Xhes6 function, a rescue experiment was performed. mRNA encoding Xhes6 that is not recognised by Xhes6 MO1 was injected into 2-cell stage embryos with or without the morpholino [33]. As reported previously, embryos injected with *Xhes6* mRNA alone showed increased expression of both *N-tubulin* and *NeuroD* generally within the neural plate at neurula stage, indicating that over expression of Xhes6 promotes neurogenesis (Fig. 2C, 2K and Table 1, [24]). This neurogenesis occurs within the usual stripes of primary neurons although some expansion of these stripes, particularly those lying most laterally, can be seen (Fig. 2C). When coinjected with Xhes6 MO1, Xhes6 mRNA restored or caused small increase in expression of *N-tubulin* within the neural plate in 51% of embryos (n = 49) and *NeuroD* in 89% of embryos (n = 36) (Fig. 2D, 2L, 2Q, 2R and Table 1). In addition, *Xhes6* mRNA occasionally induced ectopic epidermal *N-tubulin* and *NeuroD* expression beyond the normal boundary of the neural plate in some embryos (Table 1). Such expansion of the region of neural differentiation is also seen when *Xhes6* mRNA is injected without MO, and is associated with broadening of the domain of *Xngn2* expression, albeit within the normal stripes of primary neurons, leading to the hypothesis that Xhes6 promotes Xngn2 expression and function in the neural plate [24]. Taken together the MO phenotype and the results of the rescue experiments indicate that Xhes6 is required for primary neurogenesis.

**Table 1:** Effect of Xhes6 morpholinos on neural marker expression.

% embryos							
*N-tubulin in situ*							
mRNA/MO	3+	2+	1+	0	−1	−2	**n embryos**
uninjected	0	0	0	100	0	0	35
CTL	0	0	0	100	0	0	34
MO1	0	0	0	19	0	81	31
MO2	0	0	0	97	0	3	34
MO1+Xhes6	0	0	0	51	33	16	49
MO1+DBM	0	0	7	76	10	7	41
MO1+ΔWRPW	0	0	0	53	0	48	40
Xhes6	0	5	59	36	0	0	44
DBM	0	0	52	48	0	0	46
ΔWRPW	0	0	0	89	0	11	35
*NeuroD in situ*							
mRNA/MO	3+	2+	1+	0	−1	−2	**n embryos**
uninjected	0	0	0	100	0	0	54
CTL	0	0	0	100	0	0	44
MO1	0	0	0	38	0	62	39
MO2	0	0	0	95	0	5	44
MO1+Xhes6	0	0	22	67	8	3	36
MO1+DBM	0	0	11	81	8	0	37
MO1+ΔWRPW	0	0	5	61	0	34	38

Expression patterns of *N-tubulin* and *NeuroD* transcripts in embryos injected with the morpholinos and/or mRNAs shown. Appearances of typical embryos are shown in Figure 1. Scoring is as shown in Figure S2.

Previous studies have used mutant forms of Xhes6 to identify domains in the protein essential for its function. It has been reported that overexpression of a DNA-binding mutant (DBM) of Xhes6, in which the basic domain amino acids have been mutated to acidic residues, causes a similar increase in the level of *N-tubulin* positive cells to that seen with wild-type Xhes6, suggesting that Xhes6 does not need to bind DNA to promote neurogenesis [24]. We therefore investigated whether the DBM mutant could rescue the Xhes6 MO1 phenotype. Coinjection of mRNA encoding Xhes6 DBM rescued the expression of neural marker genes with the same efficiency as wild type Xhes6 mRNA, restoring a normal pattern of *N-tubulin* and *NeuroD* transcription in 76% (n = 41) and 81% (n = 37) of embryos respectively (Fig. 2F, 2N, 2Q,2R and Table 1). We went on to determine whether a ΔWRPW mutant of Xhes6 that lacks the WRPW Groucho/TLE -interaction motif could also rescue the Xhes6 MO1 phenotype [24]. In marked contrast to wild type and the DBM forms of Xhes6, injection the same 500 pg dose of mRNA encoding the ΔWRPW mutant has no effect on neural marker expression when injected on its own (Fig. 2G, 2O, 2Q, 2R and Table 1). Interestingly, however, this dose of the ΔWRPW mutant partially restored primary neurogenesis when injected together with Xhes6 MO1, resulting in essentially normal *N-tubulin* and *NeuroD* expression in 53% (n = 40) and 61% (n = 38) of embryos respectively, although the remaining embryos still often showed a marked reduction in neurons (Fig. 2H, 2P, 2Q, 2R, Table 1 and data not shown). It should be noted that higher doses of the ΔWRPW mutant result in a modest induction of neural marker expression, although significantly less than that seen with the same dose of wild type or DBM mutant Xhes6 [27]. These results indicate that Xhes6 does not need to bind DNA directly to promote neurogenesis and is still able to support neurogenesis, although to a lesser extent, when unable to recruit Groucho/TLE proteins.

### Xhes6 is required for the function of neurogenic regulatory factors

Hes6 expression is upregulated by proneural genes during neurogenesis in both mouse and *Xenopus* and the data presented above indicates that Xhes6 plays an essential role during neuronal differentiation [24]. Previous overexpression studies also indicated that Xhes6 upregulates the expression of *Xngn2* during the early stage of neurogenesis in the stripes where it is normally expressed [24]. We saw that injection of Xhes6 MO1 reduced *Xngn2* expression (in 53% of embryos, n = 61), supporting the hypothesis that *Xhes6* is not only a downstream target of Xngn2 but also acts in a positive feedback loop to sustain *Xngn2* expression (Fig. S3).

**Figure 3:**
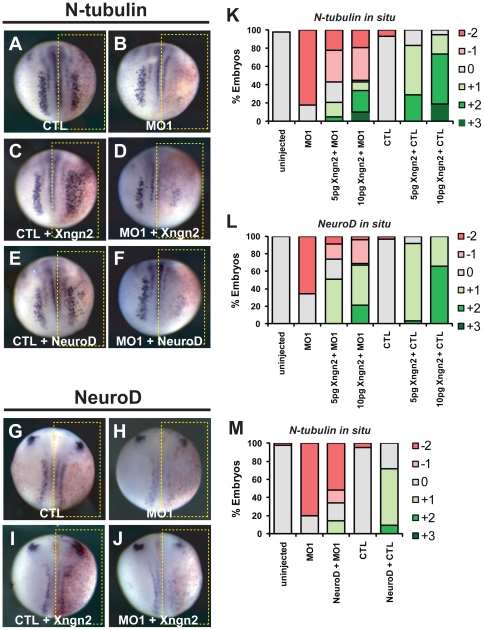
Xhes6 is required for the function of Xngn2 and NeuroD. Xngn2 or NeuroD mRNA was injected into one cell of two cell stage embryos with or without control (STD CTL) or Xhes6 (MO1) morpholinos. At neurula stage, embryos were analysed for expression of transcripts encoding N-tubulin (A−F) or NeuroD (G−J) by *in situ* hybridization. Injection of mRNAs encoding Xngn2 and NeuroD increases the number of primary neurons (C, E) but this effect is blocked by co-injection of the Xhes6 MO (D, F). Xhes6 is also required for Xngn2 to induce its target gene, *NeuroD* (J). K-L quantitation of phenotypes seen, scored as shown in Fig. S1; K, L effects of MO1 on Xngn2 induced expression of *N-tubulin* (K) and *NeuroD* (L) mRNA, M, effect of MO1 on *N-tubulin* mRNA expression induced by NeuroD. Full data on the frequency of phenotypes and the number of embryos analyzed is given in Table 2.

If Xhes6 is required to maintain normal expression of *Xngn2*, might it also be required for the function of Xngn2 protein? To examine this question, embryos were injected with Xngn2 mRNA and either the CTL MO or Xhes6 MO1 and analyzed for neural marker expression. Usually 5 pg of Xngn2 mRNA is sufficient to induce ectopic neurogenesis in the epidermis, and co-injection of this dose with the CTL MO did indeed result in differentiation of neurons expressing *N-tubulin* and *NeuroD* both within and beyond the neural plate in 83% (n = 41) and 92% (n = 38) of embyos respectively (Fig. 3C, 3I, 3K, 3L and Table 2). The ectopic expression of *N-tubulin* demonstrates the ability of Xngn2 to divert epidermal cells into the neural lineage. However when the same 5 pg dose of Xngn2 mRNA was co-injected with Xhes6 MO1, *N-tubulin* and *NeuroD* expression within the neural plate was either unchanged or substantially decreased compared with the uninjected side, although ectopic neurons in the epidermis were still seen in one third of embryos (Fig. 3D, 3J, 3K, 3L, Table 2).

**Table 2:** Activity of Xngn2 in Xhes6 morphant embryos.

% embryos							
*N-tubulin in situ*							
mRNA/MO	3+	2+	1+	0	−1	−2	**n embryos**
uninjected	0	0	0	98	0	0	66
Xhes6 MO1	0	0	0	18	0	82	66
MO1 + Xngn2 5 pg	0	5	16	22	35	22	37
MO1 + Xngn2 10 pg	10	24	9	2	36	19	58
CTL	0	0	0	93	0	7	58
CTL + Xngn2 5 pg	0	29	54	17	0	0	41
CTL + Xngn2 10 pg	19	55	21	5	0	0	58
*NeuroD in situ*							
mRNA/MO	3+	2+	1+	0	−1	−2	**n embryos**
uninjected	0	0	0	100	0	0	72
Xhes6 MO1	0	0	0	34	0	66	47
MO1 + Xngn2 5 pg	0	0	51	23	17	9	35
MO1 + Xngn2 10 pg	0	21	46	2	27	4	48
STD CTL	0	0	0	97	0	3	39
CTL + Xngn2 5 pg	0	3	89	8	0	0	38
CTL + Xngn2 10 pg	0	66	34	0	0	0	44

Table shows the expression patterns of *N-tubulin* and *NeuroD* transcripts in embryos injected with the mRNAs and/or morpholinos shown. Appearances of typical embryos are shown in Figure 2. Scoring is as shown in Figure S2.

The ability of Xhes6 MO1 to inhibit such Xngn2-mediated neurogenesis was dependent on the amount of Xngn2 mRNA injected. Co-injection of 10 pg Xngn2 mRNA with Xhes6 MO1 resulted in increased expression of *N-tubulin* and *neuroD* in approximately half of the embryos, whilst Xhes6 MO1 had no effect on neural marker expression following a 50 pg dose of Xngn2 RNA (Fig. 3K, 3L, Table 2 and data not shown). These observations are consistent with Xhes6 acting to promote the expression and/or function of Xngn2, in a dose dependent manner, both within the neural plate and in the epidermis. At high Xngn2 doses, the requirement for Xhes6 may be bypassed by an excess of Xngn2 protein, or alternatively Xngn2 induced *Xhes6* transcription may overcome the inhibitory effect of the Xhes6 morpholino [18], [24].

Given the requirement for Xhes6 for Xngn2 protein function, we went on to investigate whether Xhes6 is also required for the function of the proneurogenic *NeuroD,* a direct downstream target of Xngn2. The majority of embryos co-injected with 20 pg of *NeuroD* mRNA and CTL MO showed increased N-tubulin expression at injected side, both within the neural plate and in the epidermis (72%, n =  97, Fig. 3E, 3M, Table 3), whereas co-injection of *NeuroD* mRNA and Xhes6 MO1 resulted in the inhibition of neurogenesis in majority (67%, n = 96) of embryos (Fig. 3F, 3M, Table 3). These data indicate that Xhes6 is required for the neurogenic activity of both Xngn2 and NeuroD, both within the neural plate and for the formation of ectopic neurons in the epidermis.

**Table 3:** Activity of NeuroD in Xhes6 morphant embryos.

% embryos							
*N-tubulin in situ*							
mRNA/MO	3+	2+	1+	0	−1	−2	**n embryos**
uninjected	0	0	0	98	0	2	46
Xhes6 MO1	0	0	0	20	0	80	61
MO1 + NeuroD	0	0	14	20	15	52	96
STD CTL	0	0	0	95	0	5	59
CTL + NeuroD	0	9	63	28	0	0	97

Table shows the expression patterns of *N-tubulin* transcript in embryos injected with the morpholinos and/or mRNAs shown. Appearances of typical embryos are shown in Figure 2. Scoring is as shown in Figure S2.

**Figure 4:**
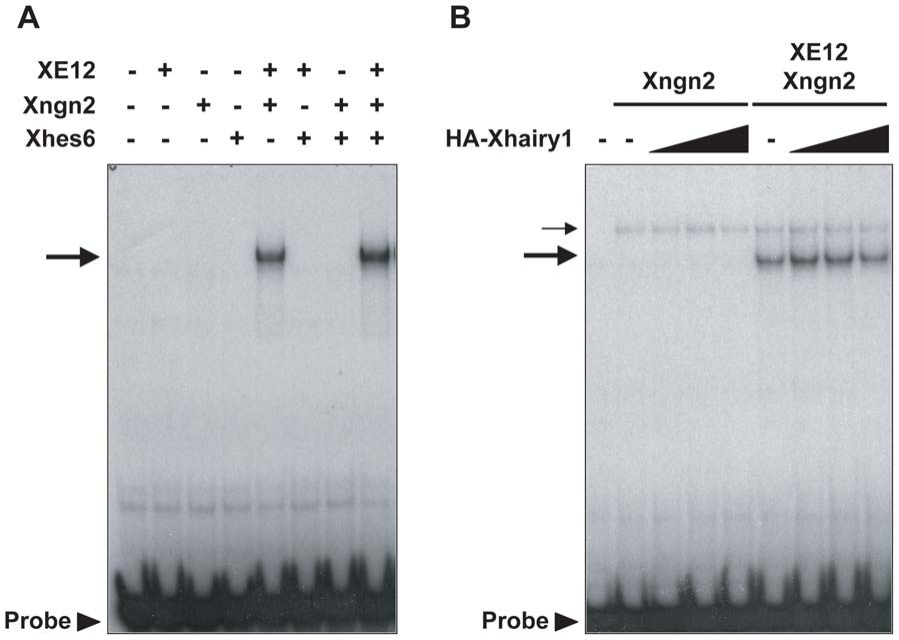
Effects of Hes6 and Xhairy1 proteins on Xngn2/E12 DNA-binding activity. A DNA fragment containing E-box element from the mouse NeuroD promoter was labeled with ^32^P and incubated with the *in vitro* translated proteins shown. The reaction mixture was then analyzed on a native polyacrylamide gel. (A) Effect of Xhes6 on DNA-binding activity of Xngn2/E12. Arrow indicates complex of Xngn2/E12 and DNA. Free probes are shown by arrowhead. (B) Effect of Xhairy1 on DNA-binding activity of Xngn2/E12. Faint bands with slow mobility (thin arrow) are non-specific protein and DNA complexes. Thick arrow indicates Xngn2/XE12 probe complex. Neither Xhes6 and Xhairy1 affect heterodimer formation or the DNA binding activity of Xngn2/XE12 proteins.

We speculated that Xhes6 may act by binding to Xngn2 and/or blocking the ability of Xngn2 to bind its E protein coactivators. However, Xhes6 had no effect on the binding of Xngn2 to E12 as assayed in an electrophoretic mobility shift assay using E-box containing probe and *in vitro* translated proteins, and there was no interaction between tagged forms of Xhes6 and Xngn2 in a co-immunoprecipitation experiment (Fig. 4 and data not shown). The presence of Xhes6 also had no effect on the stability of the Xngn2 protein in an interphase *Xenopus* egg extract *in vitro,* where Xngn2 undergoes rapid ubiquitin mediated proteolysis (Fig. S4) [34]. These observations led us to investigate whether Xhes6 regulates Xngn2 function indirectly, via interaction with other Hes family members which inhibit the expression and/or function of proneural transcription factors as has been suggested by studies in other species. Xhairy inhibits the activity of neurogenic regulatory factors.

**Figure 5:**
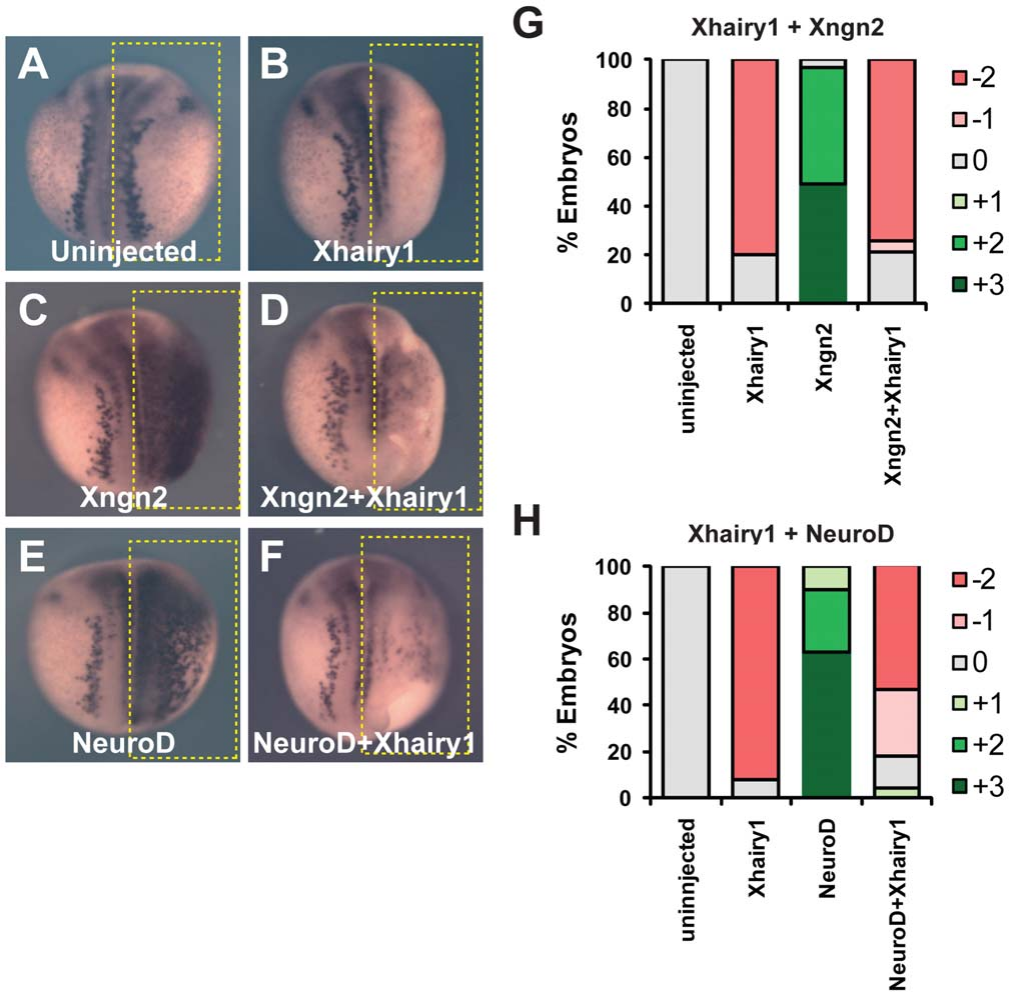
Xhairy1 inhibits the proneurogenic activity of Xngn2 and NeuroD. Embryos either uninjected (A) or with mRNAs encoding *Xhairy1* (B), *Xngn2* (C), *Xhairy1* plus *Xngn2* (D), *NeuroD* (E) or *NeuroD* plus *Xhairy1* (F) injected into one cell at the two cell stage were analyzed for *N-tubulin* expression by *in situ* hybridization at neurula stage. G,H quantitation of phenotypes seen, scored as shown in Figure S2; Full data on the frequency of phenotypes and the number of embryos analyzed is given in Tables 3, 4, and 5.

Hes1 has been shown to inhibit the expression and/or function of proneural proteins in mammals [23], [35]. In *Xenopus*, there are three homologues of Hes1, Xhairy1, Xhairy2A and Xhairy2B, all of which are expressed in the neural plate (Fig. S1, [18], [24]). We speculated that these Xhairy proteins may inhibit the function of Xngn2 and NeuroD. Overexpression of Xhairy1 or 2 by microinjection of mRNA at the 2 cell stage substantially reduced neuronal differentiation at neurula stage (Fig. 5B, 5G, 5H, Tables 4, 5, [18]). Furthermore, whilst injection of mRNA encoding Xngn2 or NeuroD produces extensive neurogenesis both within the neural plate and epidermis (Fig. 5C, 5E, 5G, 5H, Tables 4, 5), co-expression of Xhairy1 mRNA abolishes this phenotype (Fig. 5D, 5F, 5G, 5H, Tables 4, 5). We conclude that Xhairy1 powerfully inhibits the function of Xngn2 and NeuroD, consistent with the actions of homologous proteins in other species. However we see that Xhairy1 does act by not blocking the ability of Xngn2 to bind DNA or *Xenopus* E12 proteins *in vitro* (Fig. 4).

**Table 4:** Effect of Xhairy1 on Xngn2 activity.

% embryos							
*N-tubulin in situ*							
mRNA/MO	3+	2+	1+	0	−1	−2	**n embryos**
uninjected	0	0	0	100	0	0	60
Xhairy1	0	0	0	20	0	80	65
Xngn2	49	48	0	3	0	0	75
Xngn2+Xhairy1	0	0	0	21	5	74	58
*NeuroD in situ*							
mRNA/MO	3+	2+	1+	0	−1	−2	**n embryos**
uninjected	0	0	0	100	0	0	43
Xhairy1	0	0	0	33	0	67	43
Xngn2	0	96	4	0	0	0	54
Xngn2+Xhairy1	0	0	0	32	11	57	28

Table shows the expression patterns of *N-tubulin* and *NeuroD* transcripts in embryos injected with the mRNAs shown. Appearances of typical embryos are shown in Figure 3. Scoring is as shown in Figure S2.

**Table 5:** Effect of Xhairy1 on NeuroD activity.

% embryos							
*N-tubulin in situ*							
mRNA	3+	2+	1+	0	−1	−2	n embryos
uninjected	0	0	0	100	0	0	15
Xhairy1	0	0	0	8	0	92	24
NeuroD	63	27	10	0	0	0	52
NeuroD+Xhairy1	0	0	4	14	29	54	28

Table shows the expression patterns of *N-tubulin* transcript in embryos injected with the mRNAs shown. Appearances of typical embryos are shown in Figure 3. Scoring is as shown in Figure S2.

### Xhes6 and Xhairy1 antagonise each other independently of Groucho binding

**Figure 6:**
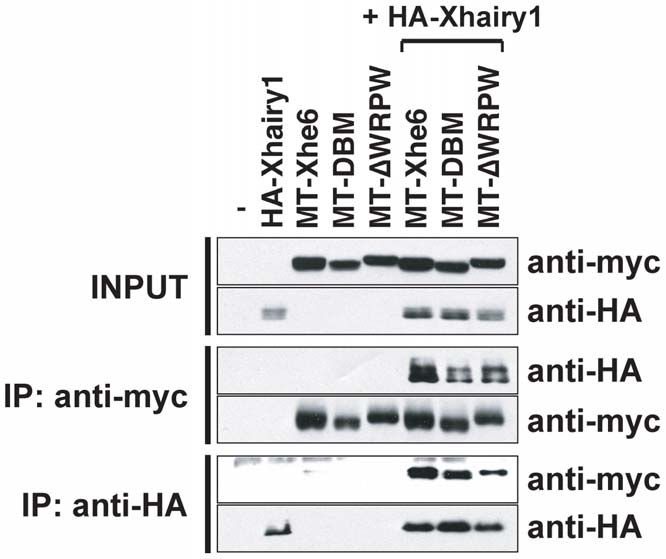
Physical Interaction of Xhes6 and Xhairy1. HA tagged Xhairy1 and myc tagged Xhes6 proteins (wild type, DNA binding mutant (DBM) and WRPW deletion mutant (ΔWRPW)) were translated *in vitro* and mixed as indicated. Following incubation with the antibody shown immunocomplexes analyzed by sodium dodecyl sulfate gel electrophoresis after which tagged proteins were detected by Western blotting. Wild type and both mutant forms of Xhes6 protein bind Xhairy1.

**Figure 7:**
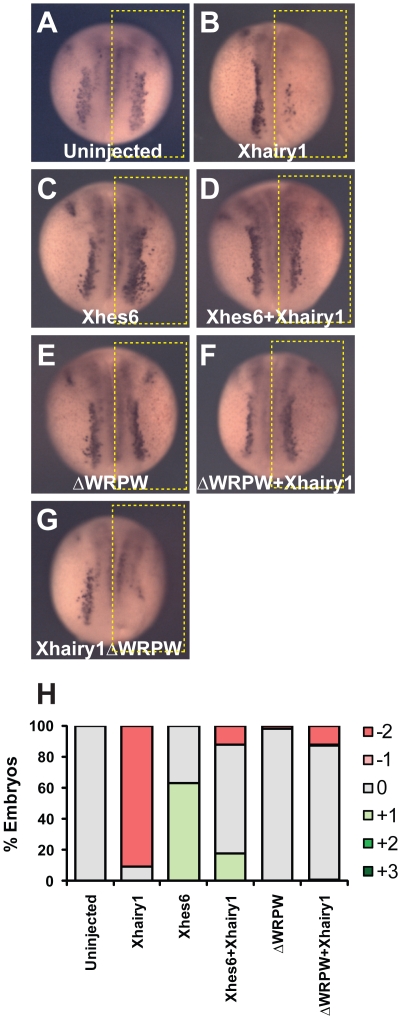
Inhibition of Xhairy1 by Xhes6 does not require the Xhes6 Groucho binding domain. Expression of *N-tubulin* transcript in uninjected embryos (A) or embryos in which mRNA encoding *Xhairy1* (B), *Xhes6* (C), *Xhairy1* plus *Xhes6* (D), *Xhes6*ΔWRPW mutant (E), *Xhairy1* plus *Xhes6*ΔWRPW (F) or Xhairy1ΔWRPW mutant (G) was injected into one cell at the two cell stage. H: quantitation of changes in *N-tubulin* mRNA expression, scored as shown in Figure S2. Full data on the frequency of phenotypes and the number of embryos analyzed is given in Table 6.

The results presented above indicate that both Xhes6 and Xhairy1 regulate the activity of Xngn2 and NeuroD. Tagged forms of Xhes6 and Xhairy1 co-immunoprecipate *in vitro* (Fig. 6). We therefore examined whether Xhes6 acts by antagonizing Xhairy1 function *in vivo*. Injection of *Xhairy1* mRNA significantly reduced the expression of *N-tubulin* in 91% of embryos (n = 43, Fig. 7B, 7H, Table 6), whereas embryos injected with *Xhes6* mRNA showed increased neurogenesis within the neural plate (63% of embryos, n = 59, Fig. 7C, 7H, Table 6). The majority of embryos coinjected with both *Xhairy1* and *Xhes6* mRNAs showed essentially normal *N-tubulin* and *NeuroD* expression (70%, n = 57, Figure 7D, Table 6, and data not shown). We conclude that Xhes6 and Xhairy1 bind directly to each other and are functionally antagonistic.

**Table 6:** Effect of Xhes6 on Xhairy1 activity.

% embryos							
*N-tubulin in situ*							
mRNA	3+	2+	1+	0	−1	−2	n embryos
Uninjected	0	0	0	100	0	0	49
Xhairy1	0	0	0	9	0	91	43
Xhes6	0	0	63	37	0	0	59
Xhes6+Xhairy1	0	0	18	70	0	12	57
ΔWRPW	0	0	0	98	0	2	65
ΔWRPW+Xhairy1	0	0	1	86	1	12	98
Xhairy1ΔWRPW	0	0	0	32	0	68	57

Expression patterns of *N-tubulin* transcript in embryos injected with the mRNAs shown. Appearances of typical embryos are shown in Figure 4. Scoring is as shown in Figure S2.

To investigate the mechanism of antagonism between Xhes6 and Xhairy1, we investigated the ΔWRPW mutants of Hes6 and Xhairy1 that lack the Groucho/TLE binding WRPW motif. Co-immunoprecipitation of tagged proteins indicates that the WRPW domain is not required for Xhes6 protein to bind Xhairy1 *in vitro* (Fig. 6). We reasoned that if the *in vivo* antagonism between Xhes6 and Xhairy1 was result of a direct interaction between the two proteins, rather than via titration of Groucho cofactors, the Xhes6 ΔWRPW mutant should be able to rescue the inhibition of neurogenesis by exogenous Xhairy1. Injection of mRNA encoding the Xhes6 ΔWRPW mutant results in no change in *N-tubulin* expression (Fig. 7E, 7H, Table 6). Strikingly, however, coinjection of mRNAs encoding Xhairy1 and ΔWRPW mutant Xhes6 restored normal *N-tubulin* expression in 86% (n = 98) of embryos (Fig. 7F, 7H Table 6). Moreover, deletion of the WRPW domain in Xhairy1 has little effect on its ability to supress neurogenesis, confirming its antineurogenic effect does not require interaction with Groucho proteins (Fig. 7G, Table 6). It should be noted, however, that alongside its inhibition of Xhairy1 function, Xhes6 requires Groucho binding ability to be fully active in promoting neurogenesis (Fig. 2H, 2P, 2Q, 2R). This argues that Xhes6 acts both in a Groucho dependent and independent manner to promote neurogenesis.

## Discussion

The data presented here show that Xhes6 is important not only for the expression of *Xngn2* and *NeuroD* but also for their function, and is required for formation of primary neurons in *Xenopus*. Our results are consistent with Xhes6 acting by at least two distinct mechanisms, resolving some of the paradoxes in the current literature. Xhes6 blocks the antineurogenic function of Xhairy1 by direct protein-protein interaction. This inhibition is independent of the ability of Hes6 to bind Groucho as the Xhes6 mutant lacking the WRPW domain both binds Xhairy1 *in vitro* and relieves the inhibition of neurogenesis by Xhairy1 *in vivo* (Figs. 6, 7). However, Xhes6 also acts in a Groucho dependent manner, as the presence of the WRPW domain is essential for full rescue of the Xhes6 morpholino phenotype. This finding is consistent with previous overexpression studies that show the WRPW deletion mutant of Xhes6 is inefficient in inducing neurons compared with wild type Xhes6 [24], [27]. As well as regulating the transcription of *Xngn2* and *NeuroD*, Xhes6 has an additional role regulating the activity of Xngn2 and NeuroD proteins. However, this functional regulation is not achieved by altering the DNA binding ability of Xngn2 or its stability (Figs. 4 and S4). Similarly there is no evidence for interaction between Xhairy1 and either the *Xenopus* E protein E12 or proneural bHLH proteins *in vitro*, which might otherwise have accounted for a requirement for Xhes6 for proneural protein function.

Our results from *Xenopus* may usefully be compared with data of the role of Hes6 in neurogenesis in other species. In the chick, two Hes6 genes have been identified, both of which are expressed in the developing neural tube, where their expression is dynamically regulated and electroporation studies indicate that they function to relieve differentiating neural progenitors from the effects of Notch signaling [28], [29]. cHes6-2 is induced early in differentiation as cells become committed to the neural lineage. It acts as a DNA bound repressor, recruiting Groucho proteins via the WRPW domain to repress transcription of the Notch effector cHes5 [29]. cHes6-1 is transiently expressed later in differentiation, in post mitotic cells which co-express proneural transcription factors, where it relieves the cHes6-2 mediated inhibition of cHes5 transcription. DNA binding domain and WRPW deletion mutants of cHes6-1 retain their ability to upregulate cHes5 expression, arguing that cHes-1 acts by directly binding other Hes proteins and inhibiting their function. The multiple mechanisms of Hes6 function described in the chick, both Groucho dependent and independent, show parallels with those we report here in *Xenopus*.

Studies of cortical progenitor cells in culture have revealed that Hes6 acts to both increase neurogenesis and inhibit gliogenesis when overexpressed [30]. In keeping with the results reported here *in vivo*, proneurogenic function of Hes6 is found to be independent of Groucho binding, although suppression of glial differentiation requires both the Groucho binding domain and the phosphorylation of conserved C terminal serine residues [31]. A range of candidate mechanisms for Hes6 activity have been proposed based on these *in vitro* studies, including interactions with Hes1 but also titration of Groucho family proteins [30]. Our observations that Xhes6 can relieve Xhairy1 mediated inhibition of neurogenesis in a Groucho independent manner, but also promote neurogenesis by a mechanism that requires Groucho binding supports these hypotheses.

Whether the single Hes6 protein found in mouse and other mammals fulfills the functions of both chick Hes6 proteins remains to be determined. To be able to act by distinct mechanisms at different stages of neural differentiation a single Hes6 protein would require regulation. In keeping with this hypothesis, mouse Hes6 contains two C terminal motifs which are subject to phosphorylation and regulate Hes6 function in vitro. These are an SDLE motif is phosphorylated by Casein Kinase 2 and an SPXXSP motif which is phosphorylated by MAP Kinase. Mutation of the SDLE motif abolishes CK2 mediated phosphorylation and decreases the proneural activity of Hes6 [25]. In contrast mutation of the SPXXSP motif has minimal effect on neuronal induction but blocks the anti astrocytic activity of Hes6 [31]. Mammalian Hes6 proteins also contain an N terminal EDED motif, not found in either chick protein, which inhibits the formation of heterodimers with Hes1 [31]. These observations argue the loss of a second Hes6 gene may be associated with increased regulation of the single Hes6 protein in mammals.

It should be noted that mice null for Hes6 appear grossly normal [24]. However a definitive characterization of these animals has not been published and whilst some aspects of Hes6 function may be redundant, it remains a possibility that Hes6 is essential for the normal differentiation of some neuronal types in mouse. It is clear that such questions are easier to study in more accessible model organisms such as *Xenopus*.

A further intriguing possibility is that Hes6 may act to alter the fluctuating expression patterns of neurogenins and Hes proteins that accompany neural differentiation. In neural progenitors, transcription of Hes1, neurogenin 2 and the Notch ligand Deltalike-1 (Dll1) oscillate [22]. On differentiation, Ngn2 and Dll1 expression are maintained at a high level and Hes1 expression is downregulated. Hes6 may play a role in the dynamic interactions between Hes1 and neurogenin that control their reciprocal oscillations, which in turn plays an essential role in progenitor maintenance.

We conclude that Hes6 is a mutltifaceted regulator of neuronal differentiation in diverse systems where it plays distinct roles both at the level of regulation of gene expression, and at the level of regulation of proneural protein function.

## Materials and Methods

### Plasmid, mRNA and *in situ* probes

Plasmids encoding Xngn2, NeuroD, Xgrg4AA, β-galactosidase and Xhes6 were described previously ([33], [34]). The Xhairy1 Image clone (4030543; BH19-d2) was purchased from Geneservice. The coding region of Xhairy1 cDNA was amplified by PCR and subcloned into pCS2+. Capped mRNA was synthesized *in vitro* from linearized plasmids using the SP6 Message Machine kit (Ambion).

### *Xenopus* embryos and injection of mRNA and morpholinos

*Xenopus laevis* embryos obtained by hormone induced laying were *in vitro* fertilized, dejellied in 2% cysteine pH8.0, and washed in 0.1x MBS. Capped mRNAs and/or morpholinos (40 ng, [33]) were injected into 2-cell stage embryos in 0.2x MBS supplemented with 4% Ficoll and 25 µg/ml Gentamicin (Gibco).

### Whole mount *in situ* hybridization

*Xenopus* embryos were fixed for 1 hr in MEMFA and stained for β-galactosidase (250 pg mRNA injected embryo) using Salmon Gal (Research Organics). Whole-mount *in situ* hybridisation was carried out as described [36] with a digoxigenin (Roche)-labeled antisense RNA probe [37], [38]. Changes in gene expression were scored in comparison with the uninjected side of the embryo. Scoring followed the scheme shown in Figure S2.

### Immunoprecipitation and Western blotting

Xhes6, its mutants and Xhairy1 were transcribed and translated in vitro using TNT SP6 Quick Coupled transcription/translation system (Promega). Immunoprecipitation and Western blotting were carried out as described previously [33].

### Protein degradation Assay

Preparation of *Xenopus* egg extracts, labeling of Xngn2 with ^35^S-methionine and degradation assays were performed as described previously [34].

### Electrophorectic mobility shift assay

All proteins were transcribed/translated in vitro as described above. E-box containing probes were designed based on the mouse NeuroD promoter sequence [39] as follows:

E1: 5′-GGACCGGGAAGACCATATGGCGCATGCC–3′,

5′-GGGCCGTACGCGGTATACCAGAAGGGCC-3′,

E3: 5′- GTCTAACTGGCGACAGATGGGCCACTTT–3′,

5′-TTCTTTCACCGGGTAGACAGCGGTCAAT-3′.

Oligonucleotides were annealed and labeled with alpha-^32^P-dCTP using Klenow fragment. Probe was incubated with protein in buffer containing 20 mM Tris-HCl pH7.4, 2 mM MgCl, 50 mM KCl, 1 mM EDTA, 10% Glycerol, 1 mM DTT and 0.05 mg/ml poly(dI-dC), and protein-DNA complexes were resolved by 5% polyacrylamide gel.

## Supporting Information

Figure S1 **Expression of Xhes6 in neurula stage embryos.** *In situ* hybridization of Xenopus embryos at neurula stage for mRNA encoding *Xhes6* (A), *Xhairy1* (B) and *Xgrg4* (C). *Xhes6* mRNA expression was detected within the region where primary neurons form.
(TIF)

Figure S2 **Scoring of neural marker phenotypes.** In situ hybridizations were scored in comparison to the un-injected side of the embryo. Criteria for each category and typical appearances of embryos in each category are shown.
(TIF)

Figure S3 **Effect of Xhes6 MO1 on the expression of Xngn2 and Xhairy1.** Embryos were injected with control (CTL, A, C) or Xhes6 morpholino (MO1) (B, D) along with β-gal tracer (red staining) and analyzed for for *Xngn2* (A, B) and *Xhairy1* (C, D) transcript at neurula stage by in situ hybridization. Injection of MO1 slightly decreases *Xngn2* expression at injected side (yellow box), but not the expression of *Xhairy1*.
(TIF)

Figure S4 **Effect of Hes proteins on stability of Xngn2 protein.** Extracts were prepared from interphase *Xenopus* eggs and supplemented with ^35^S-methionine labeled Xngn2 and the non labeled *in vitro* translated proteins shown. Samples were taken at the time points indicated and analyzed by sodium dodecyl sulfate gel electrophoresis. E12 stabilizes Xngn2 protein but Xhes6 has no effect on Xngn2 stability. The stability of Xngn2 in the presence of XE12 is not affected by Xhairy1.
(TIF)
