# Supplementary figures and images for: Hes6 Is Required for the Neurogenic Activity of Neurogenin and NeuroD
Source: PLoS One. 2011 Nov 16;6(11):e27880. doi: 10.1371/journal.pone.0027880 (PMC3218063; doi:10.1371/journal.pone.0027880)

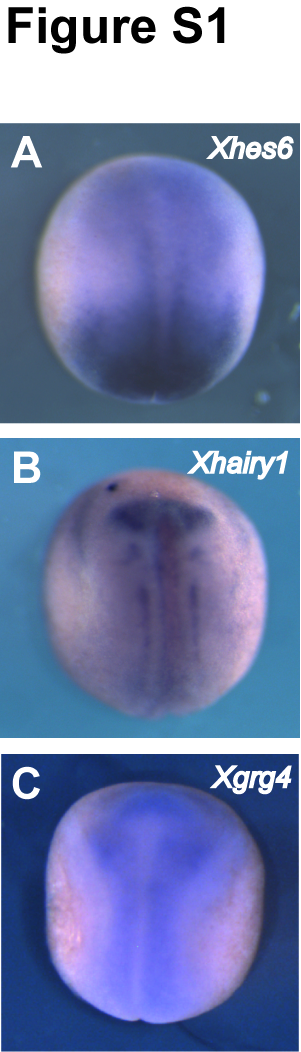

Supplement: Figure S1 — Expression of Xhes6 in neurula stage embryos. In situ hybridization of Xenopus embryos at neurula stage for mRNA encoding Xhes6 (A), Xhairy1 (B) and Xgrg4 (C). Xhes6 mRNA expression was detected within the region where primary neurons form. (TIF) [file pone.0027880.s001.tif]

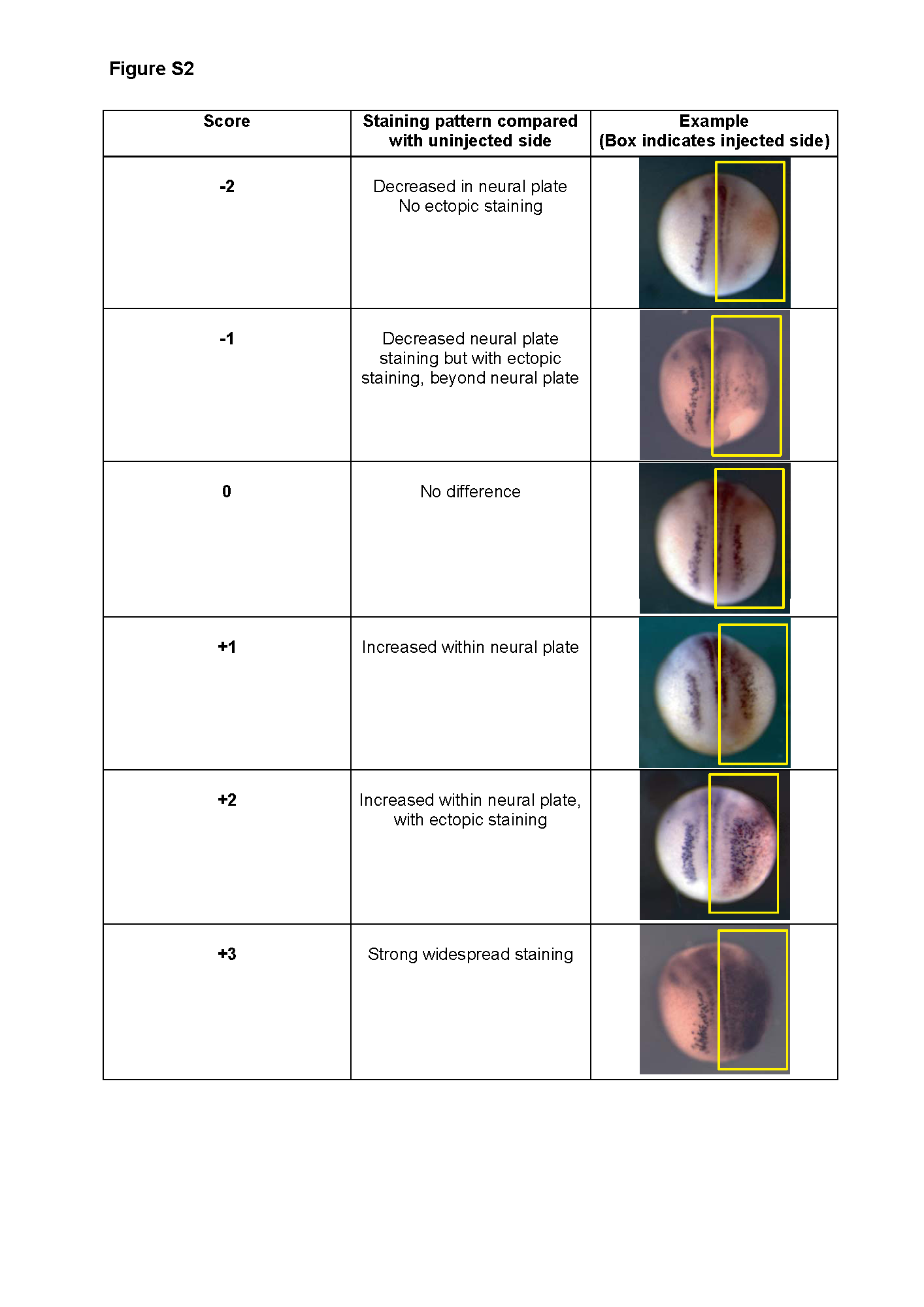

Supplement: Figure S2 — Scoring of neural marker phenotypes. In situ hybridizations were scored in comparison to the un-injected side of the embryo. Criteria for each category and typical appearances of embryos in each category are shown. (TIF) [file pone.0027880.s002.tif]

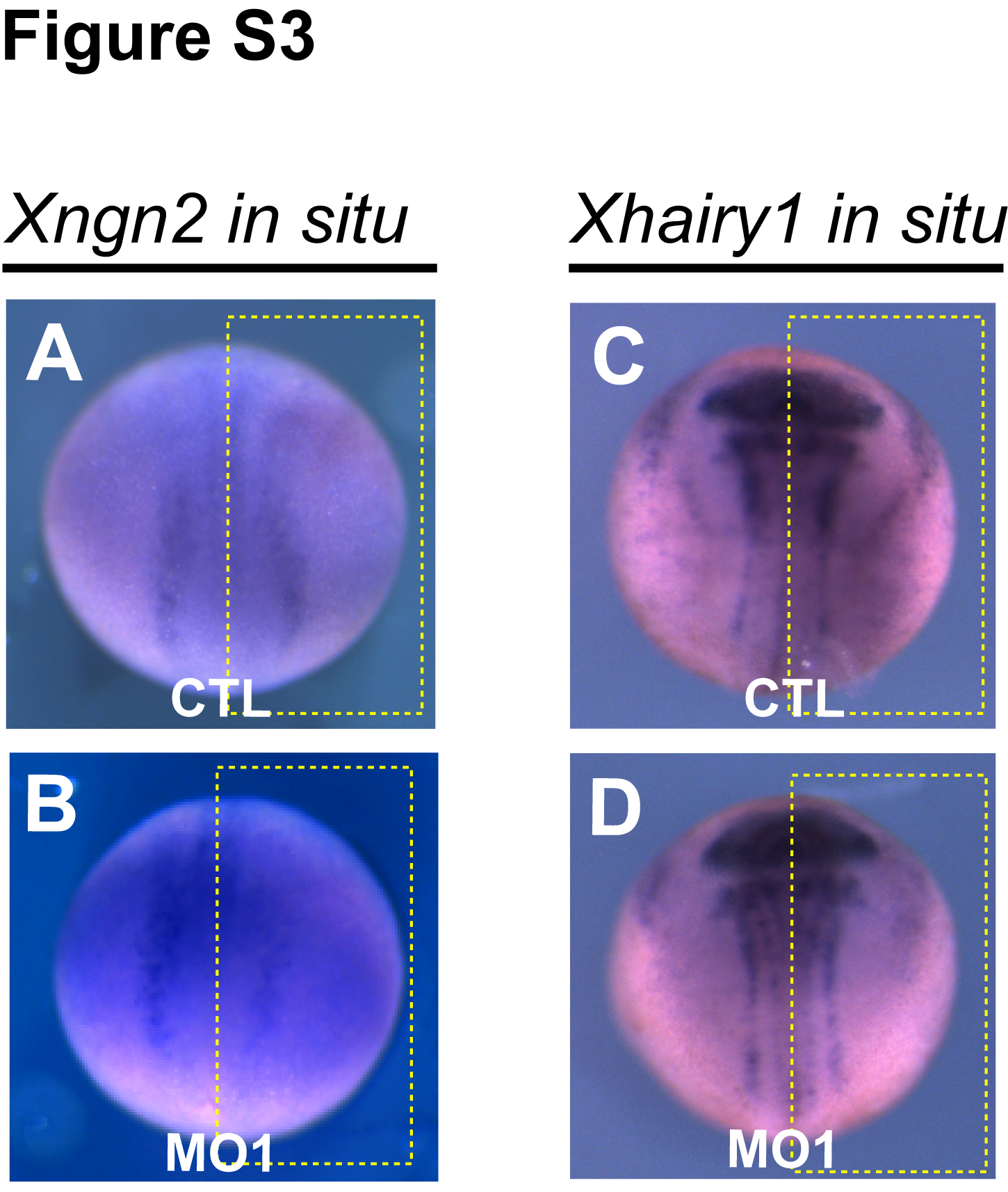

Supplement: Figure S3 — Effect of Xhes6 MO1 on the expression of Xngn2 and Xhairy1. Embryos were injected with control (CTL, A, C) or Xhes6 morpholino (MO1) (B, D) along with β-gal tracer (red staining) and analyzed for for Xngn2 (A, B) and Xhairy1 (C, D) transcript at neurula stage by in situ hybridization. Injection of MO1 slightly decreases Xngn2 expression at injected side (yellow box), but not the expression of Xhairy1. (TIF) [file pone.0027880.s003.tif]

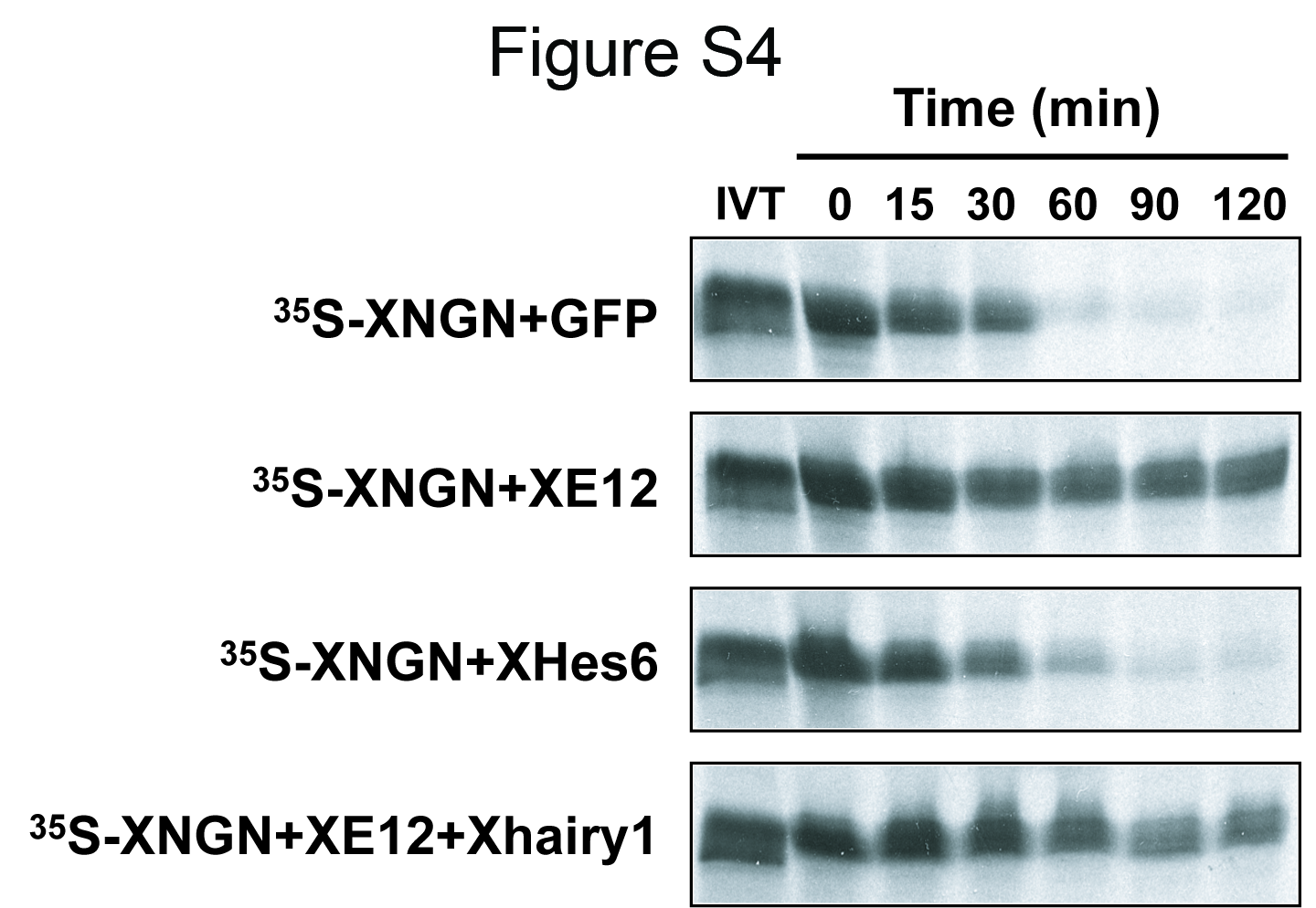

Supplement: Figure S4 — Effect of Hes proteins on stability of Xngn2 protein. Extracts were prepared from interphase Xenopus eggs and supplemented with 35S-methionine labeled Xngn2 and the non labeled in vitro translated proteins shown. Samples were taken at the time points indicated and analyzed by sodium dodecyl sulfate gel electrophoresis. E12 stabilizes Xngn2 protein but Xhes6 has no effect on Xngn2 stability. The stability of Xngn2 in the presence of XE12 is not affected by Xhairy1. (TIF) [file pone.0027880.s004.tif]
